# A review on the role of MCM3AP-AS1 in the carcinogenesis and tumor progression

**DOI:** 10.1186/s12935-022-02644-5

**Published:** 2022-07-05

**Authors:** Soudeh Ghafouri-Fard, Tayyebeh Khoshbakht, Bashdar Mahmud Hussen, Mohammad Taheri, Mohammad Samadian

**Affiliations:** 1 Department of Medical Genetics, School of Medicine, Shahid Beheshti University of Medical Sciences, Tehran, Iran; 2 Phytochemistry Research Center, Shahid Beheshti University of Medical Sciences, Tehran, Iran; 3 Department of Pharmacognosy, College of Pharmacy, Hawler Medical University, Kurdistan Region, Erbil, Iraq; 4 Center of Research and Strategic Studies, Lebanese French University, Kurdistan Region, Erbil, Iraq; 5 Institute of Human Genetics, Jena University Hospital, Jena, Germany; 6 Urology and Nephrology Research Center, Shahid Beheshti University of Medical Sciences, Tehran, Iran; 7 Skull Base Research Center, Loghman Hakim Hospital, Shahid Beheshti University of Medical Sciences, Tehran, Iran

**Keywords:** MCM3AP-AS1, lncRNA, Cancer

## Abstract

*Minichromosome Maintenance Complex Component 3 Associated Protein Antisense 1* (*MCM3AP-AS1*) is an RNA gene located on 21q22.3. The sense transcript from this locus has dual roles in the pathogenesis of solid tumors and hematological malignancies. MCM3AP-AS1 has been shown to sequester miR-194-5p, miR-876-5p, miR-543-3p, miR-28-5p, miR-93, miR-545, miR-599, miR‐193a‐5p, miR-363-5p, miR-204-5p, miR-211-5p, miR-15a, miR-708-5p, miR-138, miR-138-5p, miR-34a, miR-211, miR‐340‐5p, miR-148a, miR-195-5p and miR-126. Some cancer-related signaling pathway, namely PTEN/AKT, PI3K/AKT and ERK1/2 are influenced by this lncRNA. Cell line studies, animal studies and clinical studies have consistently reported oncogenic role of MCM3AP-AS1 in different tissues except for cervical cancer in which this lncRNA has tumor suppressor role. In the current manuscript, we collected evidence from these three sources of evidence to review the impact of MCM3AP-AS1 in the carcinogenesis.

## Introduction

Long non-coding RNAs (lncRNAs) have been vastly evaluated for their functions in the carcinogenesis [1]. These transcripts have more than 200 nucleotides and are mostly located in the nucleus [2, 3]. From a functional point of view, they can enhance or suppress establishment of transcription loops, and recruit or inhibit function of expression regulators, thus modulating transcriptional events [4–6]. In addition, they have fundamental roles in the regulation of mRNA splicing and can function as precursors for making other non-coding RNAs, particularly miRNAs [7]. The subcellular location of lncRNAs affects their functions. Within the nucleus, lncRNAs can regulate gene expression at epigenetic and transcriptional levels. In the cytoplasm, lncRNAs interact with proteins and regulate metabolism of mRNAs. Growing evidence shows that lncRNAs are imperative modulators of diverse biological processes. Through regulation of several signaling pathways, lncRNAs can act as tumor suppressive or oncogenic transcripts [8]. There are several examples of cancer-related lncRNAs in the literature. For instance, the oncofetal transcript H19 is an lncRNA which is normally expressed exclusively from the maternal allele. Yet, due to abnormalities in the imprinting, it can be up-regulated in numerous malignant tissues. Up-regulation of H19 in tumoral tissues or circulation of patients has potentiated this lncRNA as a cancer marker [9]. On the other hand, MEG3 is an lncRNA being repeatedly down-regulated in human cancers through hypermethylation of the promoter region. This lncRNA regulates expression of p53, RB, MYC and TGF-β. Moreover, it can affect epithelial-mesenchymal transition and response to chemotherapy [10]. NEAT1 is another lncRNA with fundamental roles in the establishment of paraspeckles. These nuclear sub-structures can regulate gene expression via diverse mechanisms. NEAT1 has been shown to be up-regulated in most types of cancer except for leukemia and multiple myeloma. Thus, it has been speculated that NEAT1 has distinctive roles in solid tumors and hematological malignancies [11].

*Minichromosome Maintenance Complex Component 3 Associated Protein Antisense 1* (*MCM3AP-AS1*) is an RNA gene located on chr21:46,228,977–46,259,390 (GRCh38/hg38), plus strand. The corresponding cytogenetic band is 21q22.3. The lncRNA encoded by this gene has 15 splice variants with sizes ranging from 572 bp (MCM3AP-AS1-209) to 3213 bp (MCM3AP-AS1-213) (http://asia.ensembl.org/Homo_sapiens/Gene/).

MCM3 is an important regulator in the process of DNA replication. MCM3AP is an acetyltransferase that acetylates MCM3 [12]. Up-regulation of MCM3AP suppresses DNA replication through blocking S phase entry [13]. MCM3AP contributes in the modulation of carcinogenesis process in numerous human malignant tumors [14]. In fact, MCM3AP serves as a tumor suppressive protein in breast cancer, glioma and other solid tumors [14, 15]. On the other hand, expression of MCM3AP is elevated in B-cell lymphomas and hematological malignancies [14, 16]. The antisense transcript from this locus is also involved in the pathetiology of human cancers. In the current manuscript, we collected evidence from cell line studies, investigations in xenograft models of cancers and clinical studies to review the impact of MCM3AP-AS1 in the carcinogenesis.

## Cell line studies

Cell line studies have important roles in determination of functions of lncRNAs. In these studies, the effects of up-regulation or down-regulation of a transcript on cellular functions can be assessed using apoptosis assays and migration assays. Moreover, RNA binding protein immunoprecipitation and RNA pull-down assays can be used to identify interaction between these transcripts and protein [17]. MCM3AP-AS1 silencing has blocked proliferation, colony forming ability and cell cycle progression of hepatocellular carcinoma cells, and stimulated their apoptosis. MCM3AP-AS1 has been found to interact with miR-194-5p and decrease its bioavailability, thus enhancing expression of target gene of this miRNA i.e. forkhead box A1 (FOXA1). Consistently, FOXA1 up-regulation has reversed the effect of MCM3AP-AS1 silencing on proliferation, cell cycle arrest and apoptosis [18].

**Fig. 1:**
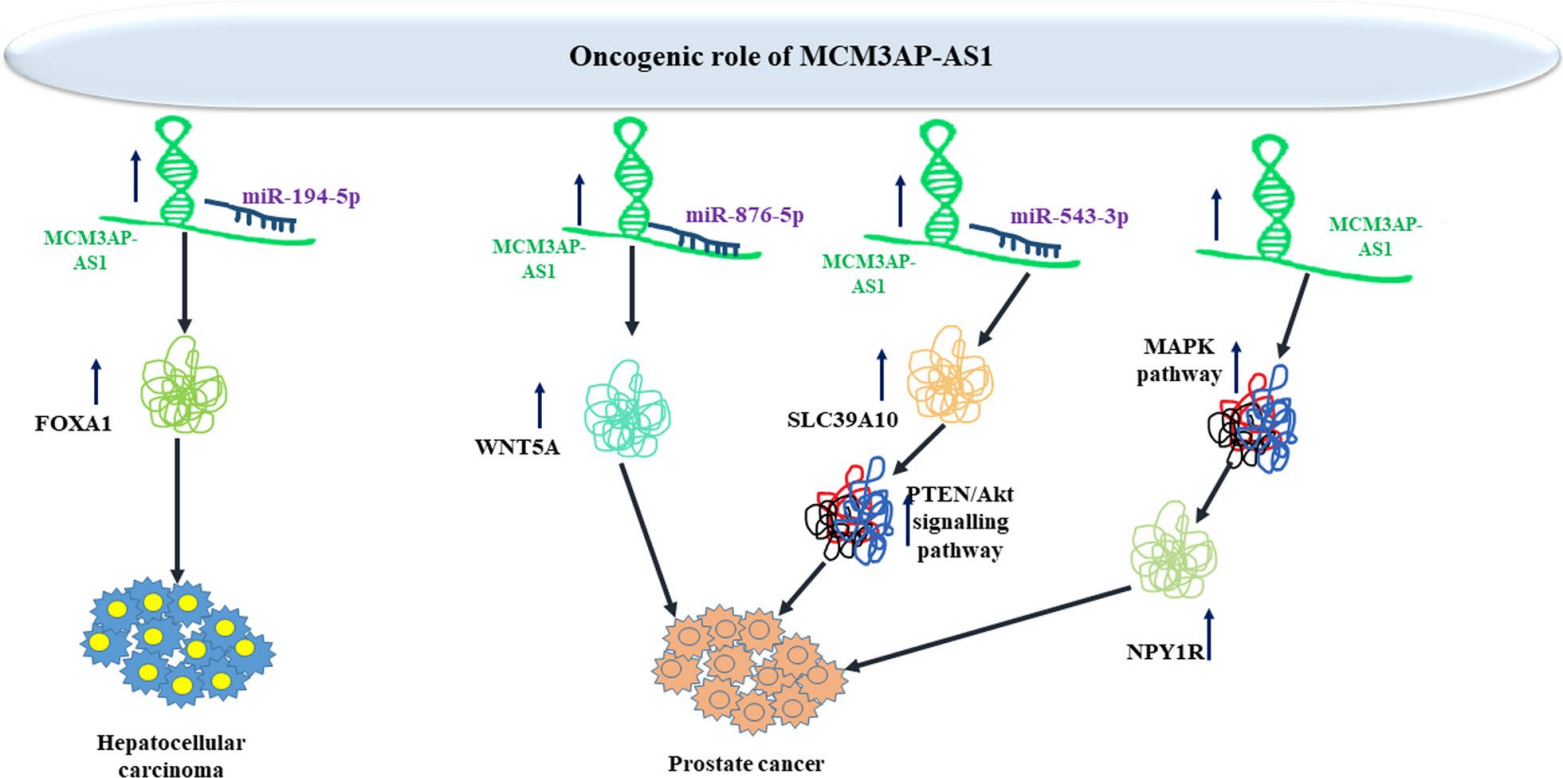
Oncogenic impact of MCM3AP-AS1 in hepatocellular carcinoma and prostate cancer

Short hairpin-mediated silencing of MCM3AP-AS1 has suppressed the proliferative capacity of prostate cancer cells and prompted their apoptosis. Mechanistically, MCM3AP-AS1 acts as a sponge of miR-876-5p to up-regulate levels of the target of this miRNA i.e. WNT5A [19]. To discover the function of MCM3AP-AS1 in prostate cancer, Wu et al. have knocked down this lncRNA in LNCaP and PC-3 cells. Based on the results of CCK-8 and EdU assays, authors have reported that proliferation of these cells has been remarkably reduced after MCM3AP-AS1 silencing. Moreover, flow cytometry technique has shown that MCM3AP-AS1 silencing increases apoptosis of these cells. Notably, expression of Bax has been increased in MCM3AP-AS1 knockdown cells, while Bcl-2 levels have been decreased, proposing that this lncRNA participates in the apoptotic processes in prostate cancer cells. These findings can also indicate that MCM3AP-AS1 is involved in the malignant features of prostate cancer [19]. Moreover, up-regulation of MCM3AP-AS1 in prostate cancer cells has been found to increase proliferation and invasive capacity through sponging miR-543-3p and influencing the SLC39A10/PTEN/Akt axis [20]. Another study in prostate cancer cells has revealed up-regulation of MCM3AP-AS1 in association with down-regulation of NPY1R. Lentivirus-mediated up-regulation of this lncRNA has increased proliferation, invasiveness, and migratory potential of prostate cancer cells while decreasing their apoptosis. Suppression of MAPK pathway has exerted opposite effects. Functionally, MCM3AP-AS1 enhances methylation of NPY1R promoter through recruiting of DNMT1/DNMT3 (A/B). Subsequent downregulation of NPY1R expression increases activity of the MAPK pathway [21]. Figure 1 depicts the oncogenic impact of MCM3AP-AS1 in liver and prostate cancers.

MCM3AP-AS1 has been found to be down-regulated in cervical squamous cell carcinoma cells. Enhancement of MCM3AP-AS1 expression has resulted in down-regulation of miR-93 and reduction of cell proliferation. Methylation-specific PCR has shown that MCM3AP-AS1 enhances methylation of miR-93 gene. Cumulatively, MCM3AP-AS1 can act as a tumor suppressor transcript in cervical cancer through decreasing miR-93 levels [22]

**Fig. 2:**
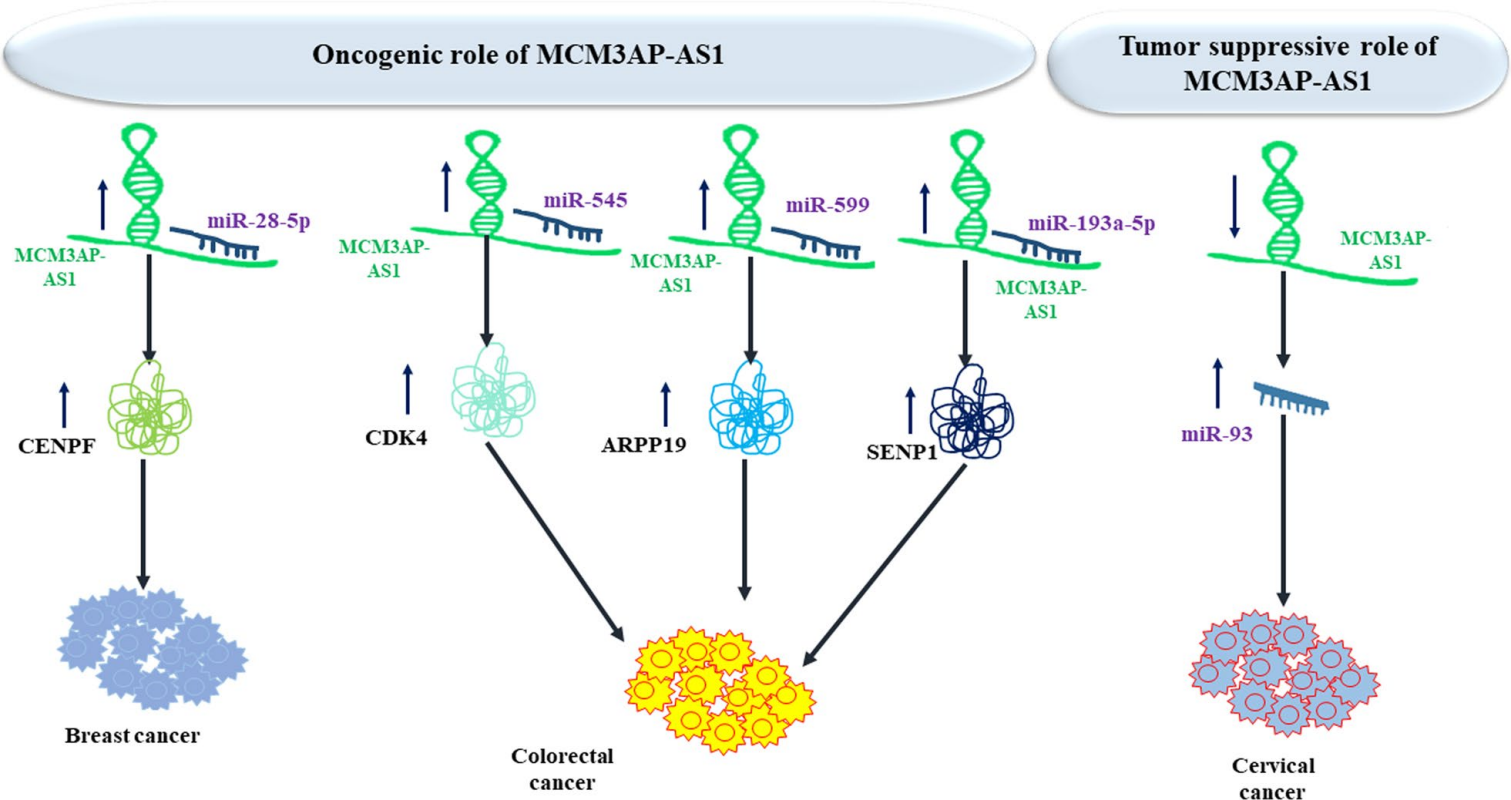
Oncogenic effect of MCM3AP-AS1 in breast and colorectal cancers and its tumor suppressor role in cervical cancer

In colorectal cancer cells, up-regulation of MCM3AP-AS1 has enhanced expression of CDK4, which is directly targeted by miR-545. Up-regulation of MCM3AP-AS1 and CDK4 has inhibited G1 arrest caused by miR-545 over-expression. Furthermore, up-regulation of MCM3AP-AS1 has decreased the facilitating impact of miR-545 up-regulation on cell cycle progression. Thus, MCM3AP-AS1 has been suggested to increase CDK4 expression through sequestering miR-545 to arrest colorectal cancer cells at G1 phase [23]. Another study in colorectal cancer cells has recognized MCM3AP-AS1 as a molecular sponge of miR-599. MCM3AP-AS1 silencing has reduced ARPP19 transcript levels and enhanced expression of miR-599. Taken together, MCM3AP-AS1 facilitates progression of colorectal cancer through affecting the miR-599/ARPP19 axis [24]. MCM3AP‐AS1 also stimulates proliferation and metastatic potential of colorectal cancer cells through regulating miR‐193a‐5p/SENP1 axis [25]. In addition, MCM3AP-AS1 enhances progression of breast cancer through affecting the activity of miR-28-5p/CENPF axis [26]. Figure 2 shows the oncogenic impact of MCM3AP-AS1 in breast and colorectal cancers and its tumor suppressor role in cervical cancer.

**Fig. 3:**
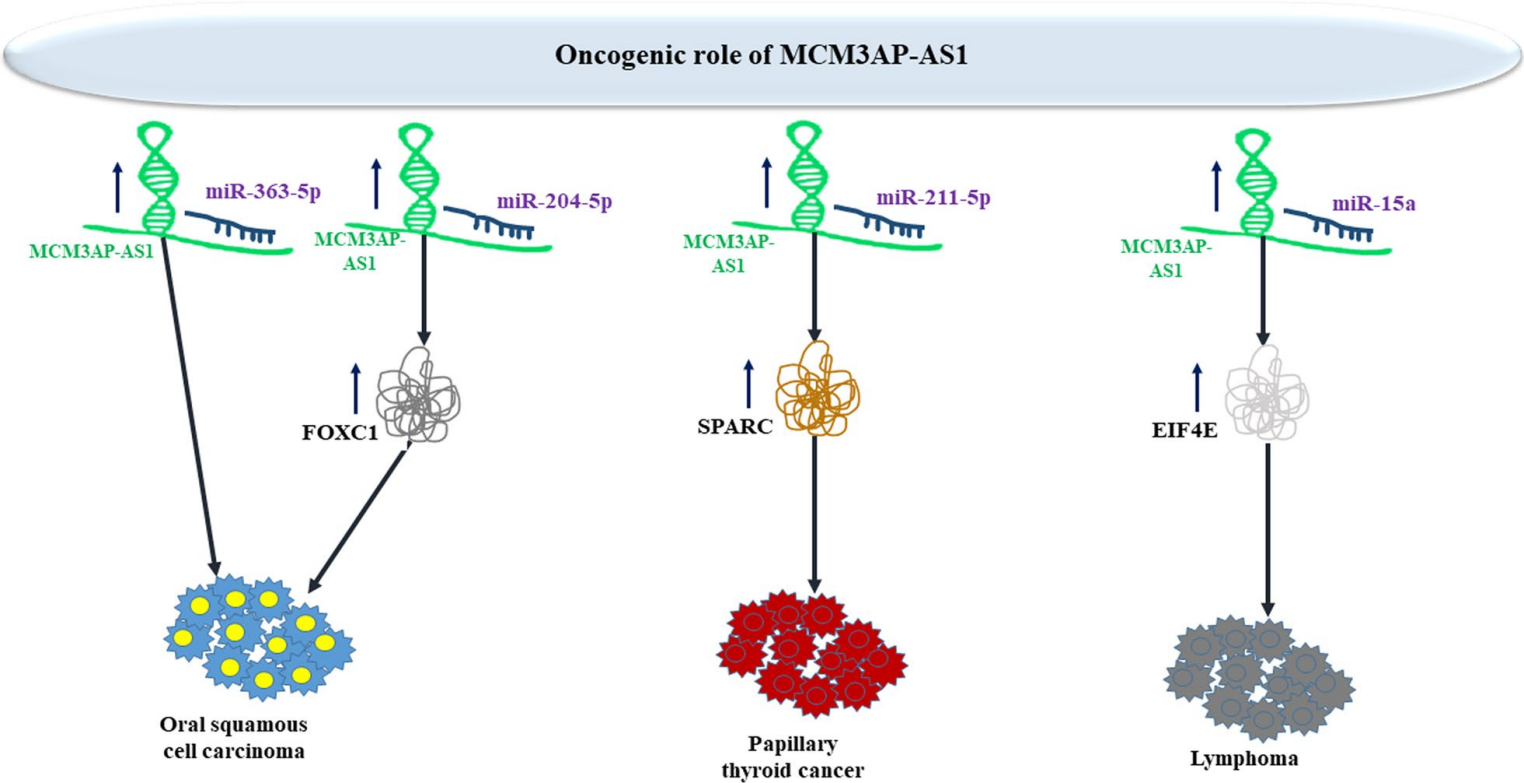
Oncogenic impact of MCM3AP-AS1 in oral squamous cell carcinoma, papillary thyroid cancer and lymphoma

Over-expression of MCM3AP-AS1 has been correlated with poor prognosis in these patients. Small interfering RNA-mediated MCM3AP‑AS1 silencing has decreased optical density value and migratory potential of oral squamous cell carcinoma cell lines. MCM3AP-AS1 has been shown to inhibit miR‑363‑5p expression [27]. miR-204-5p/FOXC1 has been identified as another molecular axis through which MCM3AP-AS1 contributes in the pathoetiology of oral squamous cell carcinoma [28]. In papillary thyroid carcinoma, MCM3AP-AS1 enhances proliferation and invasiveness via modulating miR-211-5p/SPARC axis [29]. Moreover, in lymphoma cells, MCM3AP-AS1 silencing has been shown to attenuate resistance to doxorubicin via affecting the miR-15a/EIF4E axis [30]. Figure 3 shows the oncogenic impact of MCM3AP-AS1 in oral squamous cell cancer, papillary thyroid cancer and lymphoma.

**Fig. 4:**
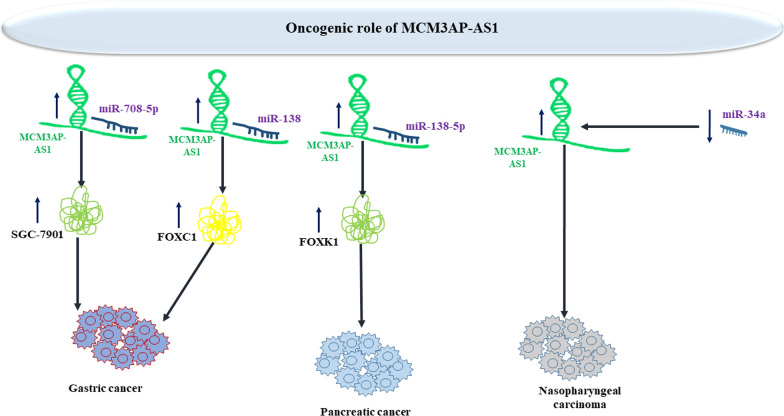
Oncogenic impact of MCM3AP-AS1 in gastric, pancreatic and nasopharyngeal cancers

MCM3AP-AS1 has been revealed to regulate proliferation and apoptosis of gastric cancer cells through regulating miR-708-5p levels [31]. Levels of MCM3AP-AS1 have been found to be higher cisplatin-resistant gastric cancer cells. MCM3AP-AS1 silencing has reduced cisplatin resistance in these cells. Mechanistically, MCM3AP-AS1 up-regulates FOXC1 levels through sequestering miR-138. Up-regulation of FOXC1 has stopped the effects of MCM3AP-AS1 silencing or miR-138 mimic on resistance to cisplatin. Taken together, MCM3AP-AS1 confers resistance to cisplatin through sponging miR-138 and up-regulating FOXC1 levels [32]. miR-138-5p/FOXK1 has been found as the molecular axis mediating the pro-proliferative effects of MCM3AP-AS1 in pancreatic cancer cells [33]. Moreover, MCM3AP-AS1 affects proliferastion and apoptosis of nasopharyngeal cancer cells through regulating expression of miR-34a [34]. Figure 4 shows oncogenic impact of MCM3AP-AS1 in gastric, pancreatic and nasopharyngeal cancers.

**Fig. 5:**
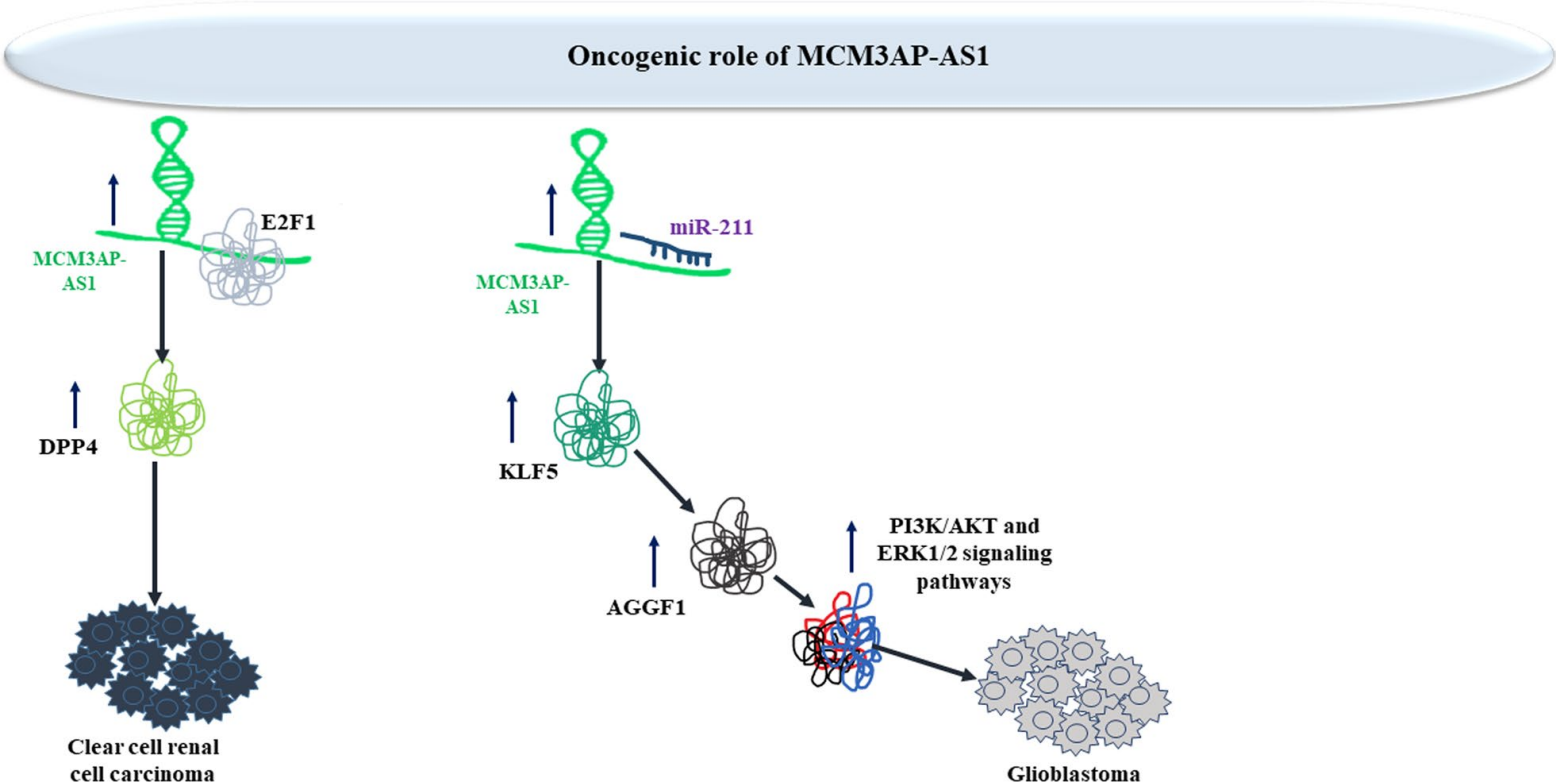
Oncogenic impact of MCM3AP-AS1 in renal cell carcinoma and glioblastoma

Assessment of methylation status of MCM3AP-AS1 in clear cell renal cell carcinoma cells has revealed demethylation of its promoter. Up-regulation of MCM3AP-AS1 has increased proliferation, production of proinflammatory cytokines, and the tube establishment of endothelial cells. MCM3AP-AS1 increases E2F1 recruitment at the DPP4 promoter to facilitate DPP4 expression. DPP4 silencing has abolished pro-angiogenic and pro-inflammatory effects of MCM3AP-AS1 in renal cancer cells [35]. In glioblastoma cells, MCM3AP-AS1 enhances angiogenesis through modulation of miR-211/KLF5/AGGF1 route [36]. Figure 5 shows oncogenic impact of MCM3AP-AS1 in renal cell carcinoma and glioblastoma.

**Fig. 6:**
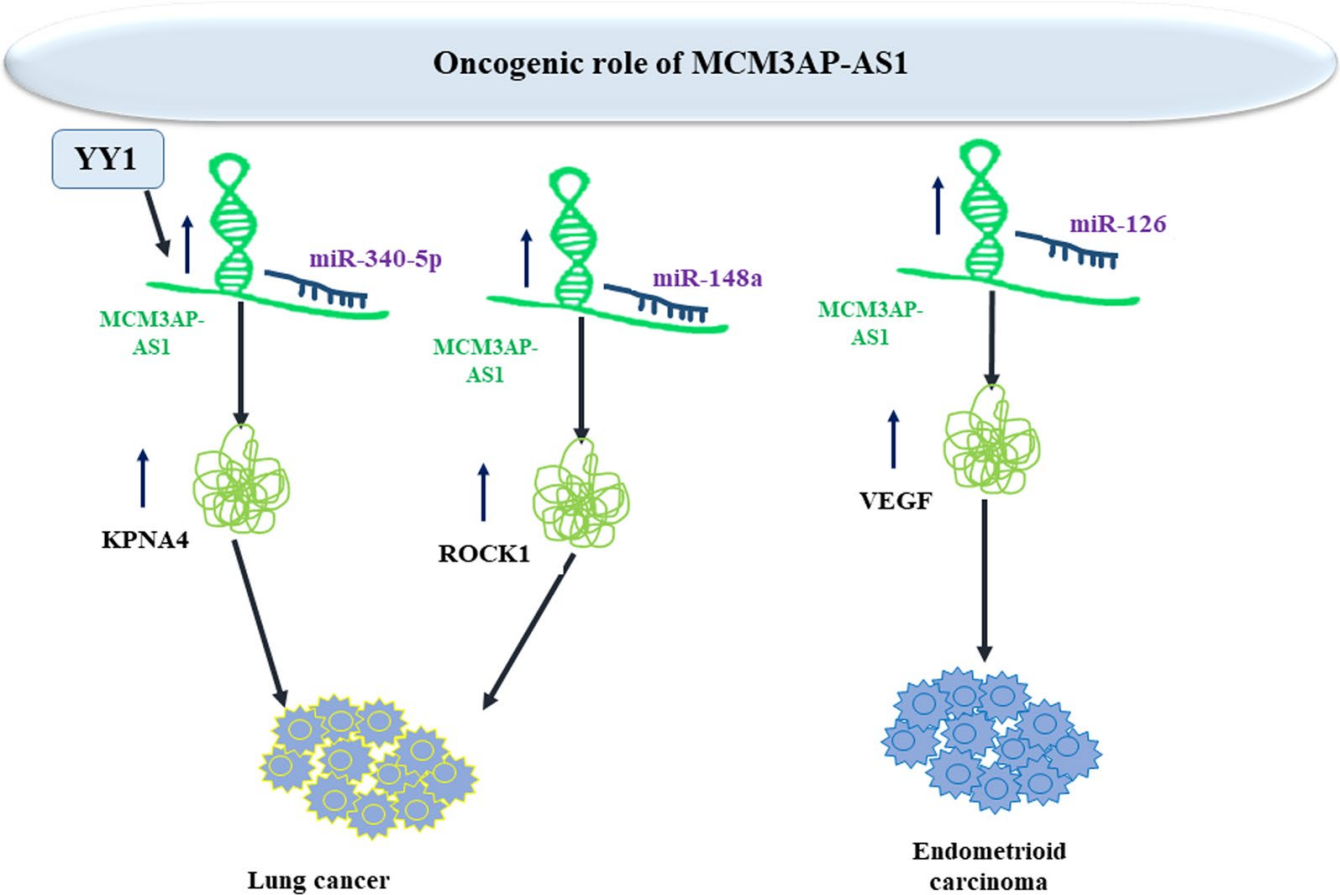
Oncogenic impact of MCM3AP-AS1 in lung and endometroid cancers

In lung cancer, transcription of MCM3AP‐AS1 is induced by YY1. Enhancement of MCM3AP‐AS1 levels enhances angiogenic process and cancer progression through influencing miR‐340‐5p/KPNA4 molecular axis [37]. Moreover, it sequesters miR-148a to increase invasiveness and migratory potential [38]. In endometrioid carcinoma, miR-126/VEGF axis mediates cancer cell invasiveness and migration [39]. Figure 6 shows oncogenic impact of MCM3AP-AS1 in lung and endometroid cancers.

Table 1 shows expression levels of MCM3AP-AS1 in cell lines.

**Table 1:** Summary of research papers appraised expression of MCM3AP-AS1 in cell lines

Tumor type	Target/ Regulator/ Signaling	Cell line	Function	References
Hepatocellular carcinoma	miR-194-5p, FOXA1	LO2, HepG2, Hep3B, Huh7, SMMC-7721	∆ MCM3AP-AS1: ↓ proliferation, ↑ cell cycle arrest, ↑ apoptosis	[18]
Prostate cancer	miR-876-5p, WNT5A	C4-2, PC-3, LNCaP, DU145, 22Rv1	∆ MCM3AP-AS1: ↓ proliferation, ↑ apoptosis	[19]
	miR-543-3p, SLC39A10, PTEN/Akt signaling pathway	PC-3, DU145, 22RV1, LNCaP, WPMY-1	∆ MCM3AP-AS1: ↓ proliferation, ↓ migration, ↓ invasion, ↑ cell cycle arrest, did not affect apoptosis	[20]
	NPY1R, MAPK pathway	RWPE1, 22RV1, LNCaP, and DU145	∆ MCM3AP-AS1: ↓ proliferation, ↓ migration, ↓ invasion, ↑ apoptosis	[21]
Breast cancer	miR-28-5p, CENPF	MCF-10A, MCF-7, BT-549, MDA-MB-468, HS578 T	∆ MCM3AP-AS1: ↓ proliferation, ↓ migration, ↓ invasion	[26]
Cervical cancer	miR-93	C-33A, SiHa	↑ MCM3AP-AS1: ↓ proliferation	[22]
Colorectal cancer	miR-545, CDK4	CR4	↑ MCM3AP-AS1: ↑ proliferation, ↓ G1 arrest	[23]
	miR-599, ARPP19	HCT-116, SW620, SW480, LoVo, NCM460, 293 T	↑ MCM3AP-AS1: ↑ proliferation, ↑ migration	[24]
	miR‐193a‐5p, SENP1	HCT‐8, HCT116, LoVo, HT29, and SW620	∆ MCM3AP-AS1: ↓ proliferation, ↓ migration, ↓ invasion	[25]
Oral squamous cell carcinoma	miR-363-5p	HOK, SCC-9, SCC-15, CAL-27	∆ MCM3AP-AS1: ↓ proliferation, ↓ migration, ↓ invasion	[27]
	miR-204-5p, FOXC1	HN-6, SCC-9	∆ MCM3AP-AS1: ↓ proliferation, ↓ migration, ↓ invasion	[28]
Papillary thyroid cancer	miR-211-5p, SPARC	TPC-1, HTH83, 8505C, SW1736, BCPAP	∆ MCM3AP-AS1: ↓ proliferation, ↓ migration, ↓ invasion	[29]
Lymphoma	miR-15a, EIF4E	Daudi, Namalwa	∆ MCM3AP-AS1: ↓ proliferation, ↑ apoptosis, ↑ sensitivity to doxorubicin	[30]
Gastric cancer	miR-708-5p, SGC-7901	GES-1, MGc-803, SGC-7901	∆ MCM3AP-AS1: ↓ proliferation, ↑ apoptosis	[31]
	miR-138, FOXC1	AGS, MKN45, NCI-N87, SNU638, GES-1, 293 T	∆ MCM3AP-AS1: ↑ CDDP sensitivity	[32]
Pancreatic cancer	miR-138-5p, FOXK1	HPDE6-C7, PANC-1, BxPC-3, MIA PaCa-2, Capan-2, AsPC-1, HEK-293	↑ MCM3AP-AS1: ↑ proliferation, ↑ migration, ↑ invasion	[33]
Nasopharyngeal carcinoma	miR-34a	FNA, C666-1, 13-9B	↑ MCM3AP-AS1: ↑ proliferation, ↓ apoptosis	[34]
Clear cell renal cell carcinoma	DPP4, E2F1	786-O, Caki-1, UT14, UT48 HK-2	↑ MCM3AP-AS1: ↑ proliferation, ↑ angiogenesis, ↑ inflammatory responses	[35]
Glioblastoma	miR-211, KLF5, AGGF1, PI3K/AKT and ERK1/2 signaling pathways	hCMEC/D3, HEK293T	∆ MCM3AP-AS1: ↓ viability, ↓ migration, ↓ tube formation, ↓ angiogenesis	[36]
Lung cancer	miR‐340‐5p, KPNA4, YY1	A549, H1299, H596, H520	∆ MCM3AP-AS1: ↓ angiogenesis, ↓ migration	[37]
	miR-148a, ROCK1	SHP-77	∆ MCM3AP-AS1: ↓ migration, ↓ invasion	[38]
	miR-195-5p, E2F3	A549, H358, H1299, H460, H226, BEAS-2B	↑ MCM3AP-AS1: ↑ proliferation, ↑ migration, ↑ invasion ↓ apoptosis	[40]
Endometrioid carcinoma	miR-126, VEGF	HEC-1	↑ MCM3AP-AS1: ↑ migration, ↑ invasion	[39]

∆: knock-down or deletion, CDDP: cisplatin

## Animal studies

Animal studies have important positions in cancer research. Animal models have been used for simulation of human body. Several animal models have been established and their application has been evaluated in cancer research. Chemical induction, xenotransplanted models and gene programmed models have been used in this field [41]. Xenotransplantation has been the most widely used model for assessment of function of MCM3AP-AS1. In this method, cancer cell lines have been manipulated to over-express or down-regulate MCM3AP-AS1. Then, these cell lines have been transplanted into animals.

To appraise the impact of MCM3AP-AS1 silencing on tumorigenesis of hepatocellular carcinoma in vivo, Wang et al. have implanted MCM3AP-AS1-silence Hep3B cells into nude mice through subcutaneous injection. They have reported that MCM3AP-AS1 silencing significantly reduces tumor growth in animals. Moreover, subcutaneous lesions produced by MCM3AP-AS1-silenced Hep3B had lower proportion of Ki-67 expressing cells compared to tumor produced by control cells [18]. In vivo experiments in xenograft models of prostate cancer has also shown that MCM3AP-AS1 silencing considerably reduces tumor volume, and decreases the ratio of Ki67-expressing cells and expression levels of SLC39A10 in lesions [20]. Another study in prostate cancer has shown that MCM3AP-AS1 over-expression facilitates cancer development in vivo, which can be inverted by up-regulation of NPY1R [21]. Additional experiments in xenograft model of renal cell cancer has confirmed pro-angiogenic and pro-inflammatory impact of MCM3AP-AS1 [35]. Table 2 shows the results of animal studies investigating the impact of MCM3AP-AS1 in tumorigenesis.

**Table 2:** Appraisal of function of MCM3AP-AS1 in animal models

Tumor Type	Animal models	Results	(type of cells injected to the mice) (type of deletion and control)	References
Hepatocellular carcinoma	12 male BALB/c nude mice (divided into two groups (*n* = 6 per group))	∆ MCM3AP-AS1: ↓ tumor growth, ↓ tumor weight	Hep3B cells were infected with sh-MCM3AP-AS1 and sh-control	[18]
Prostate cancer	5 BALB/c mice	∆ MCM3AP-AS1: ↓ tumor volumes	Cells were transfected with LV-si-MCM3AP-AS1 or LV-si-NC	[20]
Breast cancer	BALB/c nude mice	∆ MCM3AP-AS1: ↓ tumor volumes, ↓ tumor weight	MCF-7 cells were transfected with sh-NC, sh-MCM3AP-AS1#1 or sh-MCM3AP-AS1#1 + pcDNA3.1/CENPF	[26]
Colorectal cancer	10 BALB/c athymic nude mice (divided into two groups (*n* = 5 per group))	↑ MCM3AP-AS1: ↑ tumor volumes, ↑ tumor weigh	CR4 cells with overexpression of MCM3AP-AS1 and of C (mock cells)	[23]
	10 male BALB/c nude mice (divided into two groups (*n* = 5 per group))	∆ MCM3AP-AS1: ↓ tumor growth, ↓ tumor weight	LoVo cells were transfected with sh‐MCM3AP‐AS1 or sh‐Ctrl lentiviral vector	[25]
Papillary thyroid cancer	10 BALB/c nude mice (divided into two groups (*n* = 5 per group))	↑ MCM3AP-AS1: ↑ tumor volume	TPC-1 cells were transfected with lentivirus mediated sh- MCM3AP-AS1 and sh-NC	[29]
Lymphoma	24 male BALB/c nude mice (divided into four groups n = 6 per group: (Daudi-siNC, Daudi-siMCM, Namalwa-siNC and Namalwa-siMCM))	∆ MCM3AP-AS1: ↓ tumor growth	Daudi and Namalwa cells were transfected with siNC or siMCM3AP-AS1	[30]
Pancreatic cancer	24 BALB/c nude mice (divided into two groups (*n* = 12 per group))	∆ MCM3AP-AS1: ↓ tumor volumes, ↓ tumor weight, ↓ tumor growth	BxPC-3 cells were transfected with shRNA-NC and MCM3AP-AS1 shRNA-2#	[33]
Clear cell renal cell carcinoma	20 BALB/C nude mice (divided into two groups (*n* = 10 per group))	∆ MCM3AP-AS1: ↓ tumor volumes, ↓ tumor weight, ↓ angiogenesis	ccRCC cells expressing sh-MCM3AP-AS1 expression or the relevant NC	[35]
Glioblastoma	male BALB/c athymic nude mice	∆ MCM3AP-AS1 + ↑ miR-211: ↓ angiogenesis	GECs were transfected with sh-MCM3AP-AS1, pre-miR-211, sh-MCM3AP-AS1 + pre-miR-211 and control	[36]
Lung cancer	10 male BALB/c nude mice (divided into two groups (*n* = 5 per group))	↑ MCM3AP-AS1: ↑ metastasis	A549 cells with overexpression of MCM3AP-AS1 and NC	[40]

∆: knock-down or deletion

## Clinical studies

MCM3AP-AS1 has been identified as an up-regulated lncRNA in liver carcinoma samples compared to normal liver samples according to a microarray data (GSE65485). Moreover, its expression has been reported to be increased in hepatocellular carcinoma patients in another cohort in correlation with large tumor bulk, higher grade and stage of tumors and poor prognosis of cancer [18]. Analysis of TCGA data has shown higher expression of MCM3AP-AS1 in hepatocellular carcinoma samples that have higher tumor grades compared with those with low grade tumors. Besides, MCM3AP-AS1 has been shown to be over-expressed in tumors of more advanced stages compared with early stages. Using the median of expression level of MCM3AP-AS1 in tumor samples of the mentioned cohort as a cut-off value, patients have been divided into low and high MCM3AP-AS1 subgroups. Notably, high levels of this lncRNA have been correlated with larger tumor dimension, higher grade and more advanced TNM stage. Based on the results of Kaplan–Meier survival analysis, over-expression of MCM3AP-AS1 has been correlated with poor overall survival [18]. Another study on GSE65485 dataset has revealed correlation between up-regulation of MCM3AP-AS1 in liver cancer samples and shorted survival [42].

Expression of MCM3AP-AS1 has also been demonstrated to be up-regulated in prostate cancer samples in correlation with levels of WNT5A [19]. Consistent with this finding, assessment of TCGA data has confirmed up-regulation of MCM3AP-AS1 in prostate cancer tissues compared with normal tissues. Kaplan–Meier survival analysis of TCGA data has shown that over-expression of MCM3AP-AS1 is associated with shorter disease-free survival of patients, indicating that the abnormal expression of MCM3AP-AS1 can participate in the progression of prostate cancer [19]. Another study in prostate cancer has shown up-regulation of MCM3AP-AS1 cancer samples compared with healthy tissues. Long-term follow-up of these patients has shown that over-expression of this lncRNA is associated with decreased long-term survival rate of patients. Moreover, Gleason scores for N staging has been significantly different between low and high expression group [20]. An in silico analysis of GSE3868 dataset as well as TCGA data has confirmed up-regulation of this lncRNA in prostate cancer [21].

Similarly, MCM3AP-AS1 has been upregulated in colorectal cancer tissues and its over-expression has been correlated with poor survival of these patients [23]. Moreover, levels of MCM3AP-AS1 have been shown to be higher in oral squamous cell carcinoma specimens versus normal tissues [27]. MCM3AP-AS1 has been shown to be highly expressed in clear cell renal carcinoma tissues in association with poor patient survival [35].

In breast cancer samples, expression of this lncRNA has been associated with estrogen or progesterone receptor expression [43]. In endometrioid carcinoma, up-regulation of this lncRNA has been associated with poor overall and progression-free survival [39].

Contrary to these studies, MCM3AP-AS1 has been found to be down-regulated in cervical squamous cell carcinoma in correlation with poor survival [22]. Table 3 shows the levels of MCM3AP-AS1 in different cancer types and their relevance with clinical outcomes.

**Table 3:** Abnormal levels of MCM3AP-AS1 in clinical samples

Tumor type	Samples	Expression (tumor vs. Normal)	Kaplan–Meier analysis (impact of MCM3AP-AS1 up-regulation)	Univariate/multivariate cox regression	Association of MCM3AP-AS1expression with clinicopathologic characteristics	Quantification method	Normalization method	References
Hepatocellular carcinoma (HCC)	80 pairs of HCC and ANCTs (80 patients)	Up	Poorer OS	_	Advanced tumor stages, tumor size, high tumor grade, and advanced TNM stages	qRT-PCR	2^−ΔΔCt^ Method (normalized by GAPDH and U6)	[18]
	GSE65485 (50 HCC tissues and 5 ANCTs) and GSE54236	Up	_	_	_	_	_	
	GEO databases: daGSE29721, GSE40367, GSE62232, GSE36915, GSE74618	Up	Shorter OS	_	_	_	_	[42]
Prostate cancer (PCa)	30 pairs of PCa and ANCTs (30 patients)	Up	_	_	_	qRT-PCR	2^−ΔΔCt^ method (normalized by GAPDH)	[19]
	TCGA analysis: _	Up	Shorter DFS	_	_	_	_	
	64 pairs of PCa and ANCTs (64 patients)	Up	Poorer OS	_	_	qRT-PCR	2^−ΔΔCt^ method (normalized by GAPDH)	[20]
	GEO analysis: GSE3868	Up	_	_	_	_	_	[21]
	TCGA analysis:	Up	_	_	_	_	_	
	46 pairs of PCa and ANCTs (46 patients)	Up	_	_	_	qRT-PCR	2^−ΔΔCt^ method (normalized by GAPDH)	
	GEO analysis: GSE32269, GSE26964	Up	_	_	PCa bone metastasis by regulating immune microenvironment, especially M2 macrophages	_	_	[44]
	TCGA analysis: 490 PRAD patients	Up	_	_		_	_	
Breast cancer (BC)	102 pairs of BC and ANCTs (102 patients)	Up	_	_	Estrogen or progesterone receptor expression	qRT-PCR	2^−ΔΔCt^ method	[43]
Cervical cancer	64 pairs of CSCC and ANCTs (64 patients)	Down	Higher OS	_	_	qRT-PCR	2^−ΔΔCt^ method (normalized by GAPDH)	[22]
Colorectal cancer (CRC)	60 pairs of CRC and ANCTs (60 patients)	Up	Lower OS	_	_	qRT-PCR	2^−ΔΔCt^ method (normalized by GAPDH)	[23]
	30 pairs of CRC and ANCTs (30 patients)	Up	_	_	_	qRT-PCR	2^−ΔΔCt^ method (normalized by GAPDH)	[24]
	131 pairs of CRC and ANCTs (131 patients)	Up	_	_	Larger tumor size	qRT-PCR	2^−ΔΔCt^ method (normalized by β‐actin)	[25]
	TCGA database and GEO GSE21510 database	Up	_	_	_	_	_	
Oral squamous cell carcinoma (OSCC)	36 pairs of OSCC and ANCTs (36 patients)	Up	_	_	Clinical stage and lymph node metastasis	qRT-PCR	2-ΔΔCt method (normalized by U6)	[27]
Papillary thyroid cancer (PTC)	68 pairs of papillary thyroid cancer samples and ANCTs (68 patients)	Up	Lower OS	_	_	qRT-PCR	2^−ΔΔCt^ method (normalized by GAPDH and U6)	[29]
Lymphoma	41 pairs of papillary thyroid cancer tissues and ANCTs (41 patients)	Up	Lower OS	_	Tumor size, Tumor stage	qRT-PCR	_	[30]
Pancreatic cancer (PC)	86 pairs of PC and ANCTs (86 patients)	Up	Shorter OS	_	_	qRT-PCR	2^−ΔΔCt^ method (normalized by GAPDH and U6)	[33]
Nasopharyngeal carcinoma (NPC)	55 pairs of NPC and ANCTs (55 patients)	Up	Lower OS	_	_	qRT-PCR	triplicate and 2^−ΔΔCT^ method (normalized by GAPDH)	[34]
Clear cell renal cell carcinoma (ccRCC)	GEO GSE15641 database	Up	_	_	_	_	_	(35)
	78 pairs of ccRCC and ANCTs (78 patients)	Up	Shorter OS	Expression of MCM3AP-AS1 was an independent prognostic factor for ccRCC patients	Tumors size > 7 cm	qRT-PCR	Triplicate and 2^−ΔΔCT^ method (normalized by β-actin)	
Lung cancer	60 pairs of SCLC and ANCTs (60 patients)	Up	Lower OS	_	_	qRT-PCR	Triplicate and 2^−ΔΔCT^ method (normalized by GAPDH)	[38]
	63 pairs of NSCLC and ANCTs (63 patients)	Up	Lower OS	_	_	qRT-PCR	2^−ΔΔCT^ method (normalized by GAPDH)	(40)
Endometrioid carcinoma (EC)	60 pairs of EC and ANCTs (60 patients)	Up	Poor OS and PFS	_	_	qRT-PCR	2^−ΔΔCT^ method (normalized by GAPDH)	[39]

*ANCTs* adjacent non-cancerous tissues, *OS* overall survival, *DFS* disease-free survival, *TNM* tumor‐node‐metastasis, *PRAD* prostate adenocarcinoma, *SCLC* small cell lung cancer, *NSCLC* non-small cell lung cancer, *PFS* progression-free survival

## Discussion

During recent years, transcriptome analyses have revealed thousands of lncRNAs. Growing numbers of these transcripts have been found to be associated with carcinogenesis process. These cancer-related lncRNAs have been found to affect development and progression of different cancers. Several of them have been found to participate in the pathetiology of diverse cancers, thus being proposed as biomarkers for these conditions. The functional roles of several lncRNAs in the carcinogenesis have been extensively reviewed during recent years [45–50]. MCM3AP-AS1 is a tumor-promoting lncRNA in almost all tissues except for cervical tissue. Although it is transcribed from the antisense of MCM3AP, its roles in oncogenesis seems to be independent from MCM3AP. This lncRNA has been shown to sequester miR-194-5p, miR-876-5p, miR-543-3p, miR-28-5p, miR-93, miR-545, miR-599, miR‐193a‐5p, miR-363-5p, miR-204-5p, miR-211-5p, miR-15a, miR-708-5p, miR-138, miR-138-5p, miR-34a, miR-211, miR‐340‐5p, miR-148a, miR-195-5p and miR-126. Thus, the sponging role of MCM3AP-AS1 on miRNAs is the most probable mechanism of contribution of this transcript in the carcinogenesis. A number of cancer-related signaling pathways such as PTEN/AKT, PI3K/AKT and ERK1/2 are influenced by this lncRNA.

Notably, miR-138 has been found to be sponged by MCM3AP-AS1 in gastric and pancreatic cancers. Moreover, expression of FOXC1 has been shown to be regulated by MCM3AP-AS1 in oral squamous cell carcinoma and gastric cancer through modulation of expressions of miR-204-5p and miR-138, respectively. Apart from these two examples, there is no other verified example of the same miRNA targeted by MCM3AP-AS1 in different tumors or certain mRNAs being targeted by this lncRNA through different miRNAs. In addition, AKT signaling has been shown to be regulated by MCM3AP-AS1 in prostate cancer and glioblastoma. These examples provide clues for design of specific therapies for each type of cancer.

In addition to regulation of cell apoptosis, migration and invasiveness, MCM3AP-AS1 affects response of neoplastic cells to chemotherapeutic agents, namely cisplatin and doxorubicin.

MCM3AP-AS1 can also affect tumor microenvironment, since it can influence expression of VEGF, thus affecting angiogenesis. Moreover, studies in renal cell carcinoma have suggested involvement of this lncRNA in the regulation of inflammatory responses in tumor microenvironment [35]. However, the exact role of this lncRNA on immune cell cells should be investigated in future studies.

Up-regulation of MCM3AP-AS1 in cancer patients has been correlated with poor survival of these patients. Moreover, its levels have been associated with several parameters related to aggressiveness of tumors such as greater tumor size and involvement of local lymph nodes or distant organs.

Therapeutic targeting of MCM3AP-AS1 has not been investigated in clinical settings. However, there are several putative strategies for targeting lncRNAs. For instance, Antisense oligonucleotides (ASOs), CRISPR-Cas9-based methods and small molecules have been used for manipulation of gene expression. Moreover, therapeutic manipulation of the promoter region is another strategy in this regard [51]. Other strategies to target function of MCM3AP-AS1are small molecules, nanobodies, aptamers, and RNA decoys. These strategies can possibly interrupt interaction between MCM3AP-AS1 and proteins through competition or steric blockade [52]. Although sequence-based nucleic acid treatment methods are developing rapidly, several questions principally those associated with the safety and efficiency of these methods should be answered before adaptation of these techniques in the clinical settings. The tissue-specificity of MCM3AP-AS1 targeting methods is another imperative subject which can be accomplished using specific vectors that have affinity to target tissues. Development of a number of receptor-targeted adeno-associated viral vectors is an important achievement in this regard [53].

Cumulatively, MCM3AP-AS1 is an oncogenic lncRNA which facilitates oncogenesis through different routes, thus therapeutic intervention with its expression represents a possible modality for cancer treatment. However, since it has been shown to exert tumor suppressor role in cervical cancer, a context-dependent effect might exist for this lncRNA. This note should be considered in design of anticancer modalities.

## Data Availability

The analyzed data sets generated during the study are available from the corresponding author on reasonable request.
